# High-dose *N*-acetylcysteine for long-term, regular treatment of early-stage chronic obstructive pulmonary disease (GOLD I–II): study protocol for a multicenter, double-blinded, parallel-group, randomized controlled trial in China

**DOI:** 10.1186/s13063-020-04701-8

**Published:** 2020-09-11

**Authors:** Heshen Tian, Yumin Zhou, Longhui Tang, Fan Wu, Zhishan Deng, Bijia Lin, Peiyu Huang, Shaodan Wei, Dongxing Zhao, Jingping Zheng, Nanshan Zhong, Pixin Ran

**Affiliations:** State Key Laboratory of Respiratory Disease, National Clinical Research Center for Respiratory Disease, Guangzhou Institute of Respiratory Health, The First Affiliated Hospital of Guangzhou Medical University, 151 Yanjiang Xi Road, Guangzhou, Guangdong China

**Keywords:** Chronic obstructive pulmonary disease, Spirometry, Acute exacerbation, High-dose *N*-acetylcysteine, Randomized controlled trial

## Abstract

**Introduction:**

The presence of increased oxidative stress and airway inflammation has been proven in subjects with chronic obstructive pulmonary disease (COPD). Several studies have demonstrated that drugs with antioxidant and anti-inflammatory properties such as *N*-acetylcysteine (NAC) can reduce the rate of exacerbations in patients with COPD. However, the beneficial effects of NAC in early-stage COPD are minimally discussed. We are investigating whether high-dose NAC has therapeutic effects in Chinese patients with early-stage COPD.

**Method and analysis:**

A randomized, double-blinded, placebo-controlled, parallel-group, multicenter clinical trial is evaluating the efficacy and safety of NAC for the long-term treatment of patients with early-stage COPD at 24 centers in China. Subjects aged 40–80 years and recruited by physicians or researchers with special training will be randomized to either NAC 600 mg twice daily group or matching placebo group for 2 years. Measurements will include forced expiratory volume in 1 s (FEV_1_), the number of COPD exacerbations, health-related quality, and pharmacoeconomic analysis.

**Discussion:**

Currently, there are no randomized controlled trials with high-dose *N*-acetylcysteine (600 mg twice daily) for patients with mild-to-moderate COPD (GOLD I–II). We designed this multicenter randomized controlled trial (RCT) to assess the effectiveness, safety, and cost-effectiveness of long-term treatment with high-dose *N*-acetylcysteine. The results of this trial may guide clinical practice and change the standard of early COPD management.

**Trial registration:**

Chinese Clinical Trial Registry ChiCTR-IIR-17012604. Registered on 07 September 2017.

## Introduction

Chronic obstructive pulmonary disease (COPD) is a common, preventable, and treatable disease that is characterized by persistent respiratory symptoms and airflow limitation that is due to airway and/or alveolar abnormalities usually caused by significant exposure to noxious particles or gases [1]. COPD led to 3.2 million deaths in 2017 and become the third leading cause of death in the world in 2030 [2]. Acute exacerbation of COPD leads to a rapid decline of lung function, damage of health status, a decrease of exercise endurance, a high hospitalization rate, and an increase of social burden [3]. More than 70% of patients with COPD were at stage I or II which Global Initiative for Chronic Obstructive Lung Disease (GOLD) defined as early-stage COPD [1]. Their respiratory symptoms are usually mild or asymptomatic and rarely seek medical attention before the disease worsens [4, 5]. Recent studies have found that the annual decrease rate of forced expiratory volume in 1 second (FEV_1_) in patients with early COPD is higher than that in patients with severe COPD (GOLD stages III–IV) [6, 7]. Effective prevention and treatment have been explored for earlier stages of COPD. Our previous result also proved that early treatment of COPD with tiotropium or NAC resulted in a significant health benefit, delaying the annual decline of FEV_1_ or reducing COPD exacerbation rate [8, 9]. Recently, scholars have shown increasing interest in the potential mechanism and economic burden of COPD. Therefore, it is necessary to further study its pathogenesis and seek alternative treatment modalities.

Several factors such as chronic inflammation, mucus hypersecretion, and airway oxidative stress play an important role in the occurrence and development of COPD, leading to the loss of adhesion between alveoli and small airway and the decrease of lung elasticity [1]. Therefore, drugs with antioxidant, anti-inflammatory, and mucolytic characteristics may be helpful in the treatment of early-stage COPD.

*N*-Acetylcysteine (NAC) is a mucus soluble derivative of amino acid l-cysteine, which can reduce the viscosity of airway secretions and increase the ciliary clearance rate, but it has a more potent effect in antioxidant and inflammatory regulation [10, 11]. In vivo and in vitro studies extensively proved that NAC has both direct/indirect antioxidant and anti-inflammatory properties [12–14]. Furthermore, the convenience of oral administration together with its low price may be suitable in the long-term treatment of COPD patients in less-developed areas. The long-term efficacy and safety of NAC have also been demonstrated in some lung diseases such as idiopathic pulmonary fibrosis and cystic fibrosis [15, 16]. Several studies have shown that treatment with NAC (400–1200 mg per day) reduced rates of acute COPD exacerbations [17, 18], improved pulmonary function [19, 20], and prevent hospital readmissions [21, 22]. We also noted in our previous studies that the drug can significantly reduce the acute exacerbation rate of patients with moderate COPD patients (GOLD II), but has little effect in reducing the acute exacerbation of patients with severe COPD (GOLD III), suggesting that *N*-acetylcysteine may play a more important role in the early-stage of COPD [9].

However, (1) with these inconsistencies of the results above, the effect of NAC in COPD is still indetermination; (2) the majority of these studies recruited moderate-to-severe COPD patients and had a relatively short follow-up period, a design only including early-stage COPD with long-term follow-up period should be carried through; (3) the pharmacological effect of *N*-acetylcysteine was dose-dependent, some 400–800 mg/day trials did not achieve significant effects in reducing the acute exacerbation rate and annual FEV_1_ decline rate. Thus, we designed a 2-year trial titled, “High-dose N-acetylcysteine for long-term, regular treatment of early-stage chronic obstructive pulmonary disease (GOLD I-II) in China” to investigate the efficacy and safety of 1200 mg/daily *N*-acetylcysteine in lung function, quality of life, and number and duration of acute exacerbations of COPD patients.

## Methods and analysis

### Study design

**Fig. 1:**
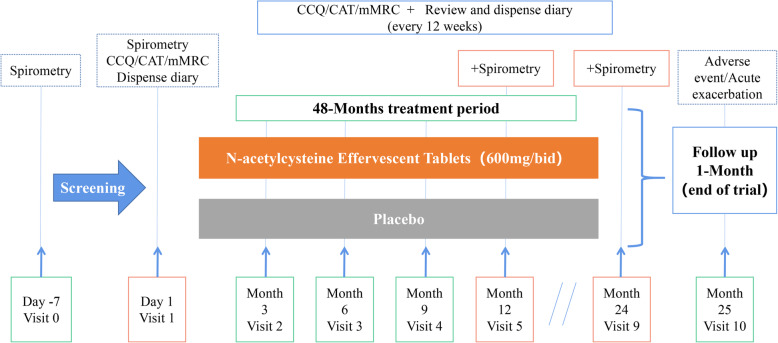
Clinical trial design. COPD, chronic obstructive pulmonary disease; CAT, COPD assessment test; CCQ, COPD clinical questionnaire; mMRC, modified British Medical Research Council dyspnea scale

This is a 2-year, randomized, double-blinded, placebo-controlled, parallel-group, multicenter trial of longitudinal treatment with high-dose NAC (600 mg twice daily) for patients with early-stage COPD. After a 1-week run-in period, 1000 patients with mild-to-moderate COPD will be randomly assigned in a 1:1 ratio to treatment with either NAC 600 mg twice daily or matching placebo for 2 years. Measurement will be scheduled for all patients at baseline and at follow-up, every 3 months for 2 years, including exacerbation rates, the modified British Medical Research Council dyspnea scale (mMRC), the COPD assessment test (CAT), the COPD clinical questionnaire (CCQ), physical variables, adverse events, medication administration, smoking status, and medical expenses. Pulmonary function tests will be conducted at the baseline and every 12 months thereafter. Figure 1 presents a flowchart of the trial procedures.

**Fig. 2:**
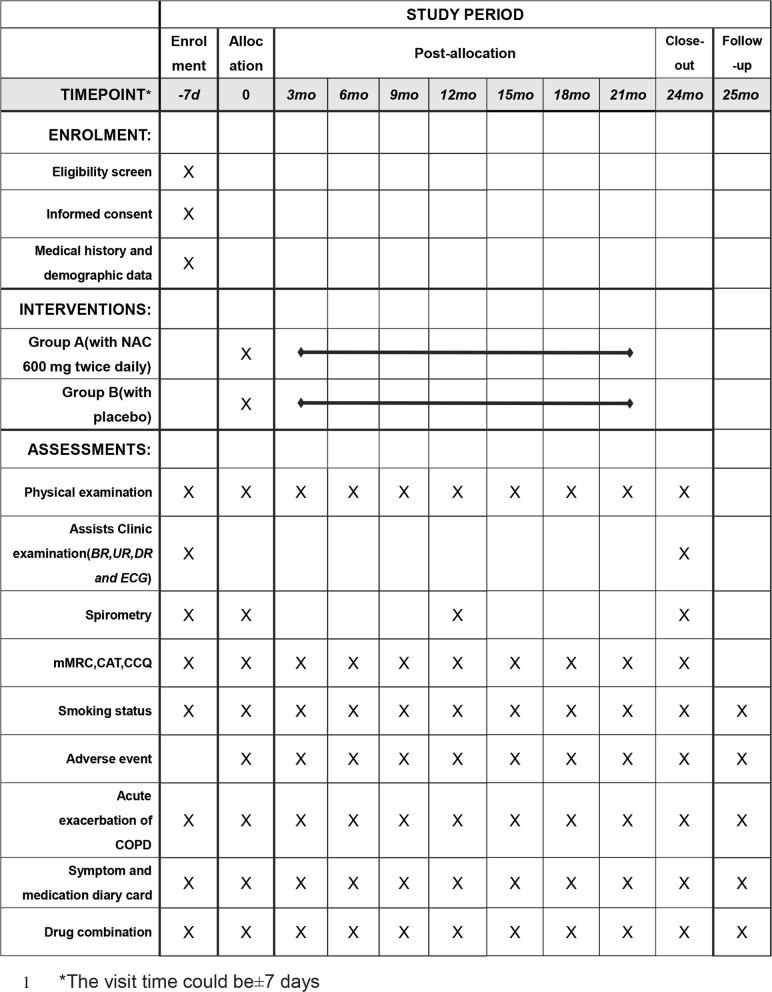
Summary of enrolment, interventions, assessments, and other activities at each study visit

The primary endpoint includes the difference of FEV_1_ (including trough and peak) at 24 months from baseline and the number of acute exacerbations of COPD within 24 months between the two groups (mean values will be used in aggregation for the primary outcome data). Secondary endpoints include the following: trough and peak FEV_1_ at 12 months; the annual decline of FEV_1_, forced vital capacity (FVC), and FEV_1_/FVC (including trough and peak); duration, interval, and severity of COPD exacerbation; time to the first COPD exacerbation; quality of life (mMRC, CAT, and CCQ); medication administration and the use of rescue medications; and adverse events (Fig. 2).

### Recruitment and population

A total of 1000 subjects from 24 study sites in China with early-stage COPD (GOLD I and II) will be recruited. Half of the subjects will be screened from the rural communities, which are advertised for general population screening with a respiratory questionnaire and simple spirometry. The rest of them will be screened from the medical centers, which are outpatients with respiratory complaints. The majority of the subjects will be symptom-free or had very slight symptoms.

Subjects will be eligible for inclusion in this study only if all of the following criteria apply: (1) aged 40–80 years old, male or female, and community or outpatient; (2) has respiratory symptoms (chronic cough, sputum, or shortness of breath) and/or a history of exposure to risk factors (smoking, occupational exposure, indoor and outdoor air pollution, etc.); (3) clinical diagnosis of COPD (GOLD stages I and II): FEV_1_/FVC < 70% and FEV_1_ ≥ 50% predicted after 20 min with 400 μg of salbutamol inhalation (Ventolin, Glaxo Wellcome); (4) patients in a stable period, that is, near 4 weeks without COPD acute exacerbation; (5) the patients are able to communicate in words, agree, and have the ability to complete the test-related auxiliary examination; and (6) sign informed consent.

Subjects will be excluded for any of the following reasons: (1) suffering from major disease (except previously known COPD) such as severe heart, brain, liver, kidney, and blood system diseases, malignant tumors, and life-threatening pulmonary embolism, affect the study completed; (2) clinical diagnosis of lung cancer, pneumoconiosis, bronchiectasis, interstitial lung disease, bronchial asthma, active tuberculosisor, alpha1-antitrypsin deficiency, cystic fibrosis, or other simple restrictive ventilation dysfunction, affect the study outcome; (3) patients with contraindications such as allergic history, intolerance to the test drug, and suffering from severe gastric ulcer or small intestine malabsorption; (4) having chronic alcohol abuse, drug abuse, or any factors that affect compliance; (5) patients who have had undergone pneumonectomy; (6) subjects who are pediatric and pregnant or would be pregnant and breastfeeding; (7) suffering from COPD acute exacerbation within 4 weeks prior to the first visit, or hospitalization and/or antibiotic treatment and/or oral or intravenous therapy were required during the enrollment; (8) those who receive NAC for more than 3 months; (9) need long-term use of inhaled glucocorticoids, systemic (oral or intravenous) hormone dose instability (i.e., dose stabilization time is less than 6 weeks) or hormone dose exceeds the equivalent of prednisone 10 mg/day, or long-term use of antibiotics;(10) patients plan to receive long-term use of oxygen therapy or rehabilitation treatment in the next 2 years; and (11) the patient is currently participating in other clinical trials.

Trial center requirements: we do not have special requirements beyond the capacity of normal medical services, but the facilities should include spirometry instrument and chest X-ray/computed tomography (CT).

### Randomization and masking

The experiment was conducted by block randomization; the blind code was generated by a statistician from Rundo International Pharmaceuticals Research & Development Co, Ltd. using SAS V.9.2.2. After 1 week of the running-in period, eligible subjects were randomly assigned to the NAC group (one 600 mg tablet twice daily) or placebo (one tablet twice daily) for 2 years in a ratio of 1:1 by an independent statistician. In other research centers, participants were assigned to *N*-acetylcysteine or placebo according to their assigned numbers. The person analyzing the data will be blinded to the allocation.

NAC and placebo tablets are manufactured and packaged by Zhejiang Jinhua CONBA Bio-pharm. Co., Ltd. The placebo was packaged to be identical in composition, shape, color, and size to NAC tablets, but it did not contain any active ingredients. To prevent serious adverse events or other emergencies, each research center keeps a confidential envelope containing the information of uncovering blindness. If the envelope is used, it should be reported.

### Concomitant medication and treatment

In principle, long-term use of concomitant medication (define as continuous medication use for more than 1 month) should be avoided during the entire visit phase because the majority of enrolled subjects have extremely slight symptoms. However, individual therapy initiated before recruitment or clinical necessary treatment will be allowed. Medical intervention according to GOLD guidelines could be applied for less than 2 weeks if patients experienced a moderate or severe exacerbation.

### Measures

Regular visit with physical examination and symptom score documentation will be conducted after the first visit and then once every 3 months thereafter. Subjects will be asked details regarding whether they have health-related adverse events in the last 3 months, receive hospitalization or outpatient and treatment, experienced any acute exacerbation, and taken any medication (rescue drugs, antitussives, expectorants, inhaled corticosteroids/oral corticosteroids or antibiotics) at each visit. Adverse drug effects, self-reported smoking status, recent changes in current medication use, and any abnormal observations will be recorded in the case report form (CRF). We will issue a follow-up diary to the patient at each follow-up and check whether it is recorded on time at the next follow-up. All of the health visits, monitor method, and health education will be the same in both arms.

### Spirometry

Baseline and post-bronchodilator lung function will be assessed at follow-up visits at 12 and 24 months by measuring FEV_1_, FVC, FEV_1_/FVC, and FEV_1_% of the predicted value. In principle, each test will be conducted at the same time (± 2 h) in the morning. Spirometers should be standardized according to the American Thoracic Society (ATS) and European Respiratory Society (ERS) criteria [23]. For the same patient, each test will be performed by the same technician and the same equipment at each research center [23]. Spirometry will be performed before and after bronchodilation (salbutamol 400 μg will be inhaled 20 min prior to conducting reversibility testing). We request at least three acceptable efforts for each subject, up to eight forced expiratory manoeuvers. The optimal value of FEV_1_ and the optimal value of FVC in any of the three acceptable results (met the ATS/ERS criteria) will be used for data statistics and outcome analysis [23].

### Exacerbations of COPD

Patients have been requested to record their exacerbation information in the patient diary and collected at every follow-up. Acute exacerbation of COPD is considered to be due to exacerbation of respiratory symptoms (cough, expectoration, expectoration, wheezing, dyspnea, at least two new symptoms or aggravation of the original symptoms, and lasting for at least 48 h). Meanwhile, the following diseases should be ruled out: left or right ventricular dysfunction, pulmonary embolism, pneumothorax, pleural effusion, and arrhythmia [1, 24]. The duration of COPD exacerbation was defined as the date from the onset of acute exacerbation to the end of the event. The duration of hospitalization was defined as the time from admission to that of discharge from the hospital. The interval between the two times of COPD exacerbation was defined as the duration between the previous exacerbation event and the next event, and it should be noted that if the interval between two events was less than 7 days, they were regarded as the same event [1].

The severity of COPD exacerbation was categorized as mild, moderate, and severe as follows: mild, adding other common medications for COPD without outpatient hospital visit or hospitalization; moderate, exacerbation requiring outpatient or emergency room visit and the use of antibiotics and/or systemic glucocorticoids; or severe acute exacerbation requiring hospitalization [25]. Specifically, the date of each exacerbation and admission are being obtained, and the times to first exacerbation and admission will be calculated.

### Quality of life

Quality of life will be assessed at all outpatient visits using designated patient record questionnaires (CAT and CCQ) [26, 27]. The mMRC dyspnea score will be recorded at every visit.

### Recruitment strategy and quality control

As the incidence rate of chronic obstructive pulmonary disease is 13.7% in people over 40 years and over 70% of them are GOLD I–II in China, so we plan to screen 10,000 people in rural areas of Guangdong province within a year until half the target population is achieved. The other centers will screen subjects to achieve the rest of the population in urban areas (21 subjects/site).

Following applicable regulations and procedures, the monitor at each center is responsible for the inspection of compliance to the protocol in terms of study conduction and accurate and timely data items, for which the CRF will serve as the source document. The monitor will contact the site prior to the start of the study to review with the site staff the protocol, study requirements, and responsibilities of the staff to satisfy regulatory, ethical, and NAC requirements. We promise that the data of this trial is true, accurate, and complete, and the safety and rights of the subjects will be protected; the relevant researchers and the person in charge of the research center are authorized to use the spirometer, share the data, and consult all relevant documents. Good compliance is defined as the amount of drug used exceeds 80% of the predicted dose of the study drug.

### Informed consent

A trained research physician or investigator will introduce the trial to the patients and inform the patients of the details of the trial drug, including pharmacological components and adverse reactions. At the same time, informed consent in written form will be provided for patients to read. Only when patients fully understand the process of the trial, they can choose to participate voluntarily or give up. Subjects who are disabled in reading or writing should be informed through their guardians/caregivers and sign informed consent on behalf of them.

### Sample size and statistical methods

We determined the sample size with regard to the primary endpoint according to the PANTHEON study [9]. The sample size was calculated based on the difference between the peak and trough values of FEV_1_ in two groups: patients with mild-to-moderate COPD were included in this study. According to the early clinical trials, FEV_1_ of GOLD II COPD patients in the experimental group and the control group after 2 years were compared. The difference in valley value was 100 ml (standard deviation was 450 ml). Assuming that the test water is 5% and the test efficiency is 80%, each treatment group needs 318 patients to detect the difference in FEV_1_ trough between the two groups. Assuming a 35% drop-off rate, each group needs to be randomized at least 489 patients. Therefore, the overall sample size of this study needs 1000 patients.

Repeated measurement analysis of variance will be used to analyze the valley and peak value of FEV_1_, CCQ, and CAT data assessments. The annual decline rate of FEV_1_, FVC, and FEV_1_/FVC was analyzed by the random coefficient regression model. Assuming that the treatment effect changes linearly with the treatment time, the annual decline rate is represented by the regression coefficient of the model. The number and degree of acute exacerbation were compared by adjusted treatment exposure dose and over discrete Poisson regression. A log-rank test was used to compare the interval time of the first exacerbation of COPD. Fisher’s exact probability method was used to analyze the interval and duration of acute exacerbation of COPD, severity, and the use of rescue drugs. The frequency of rescue drugs will be further described. Before locking the database, the analysis plan will be explained in detail in a separately prepared statistical analysis plan (SAP).

### Ethics and dissemination

This study was approved by the Ethics Committee of The First Affiliated Hospital of Guangzhou Medical University. Recruitment into this clinical trial started in September 2017 with a targeted completion date of August 2019. The follow-up of NAC treatment is currently ongoing, and the last trial visit of the last participants is scheduled to occur in September 2021 (Table 1). The study findings will be presented at conferences and reported in peer-reviewed journals.

**Table 1:** Trial registration data

Data category	Information
Primary registry and trial identifying number	Chinese Clinical Trial Registry: ChiCTR-IIR-17012604
Date of registration in primary registry	07 September 2017
Secondary identifying numbers	NA
Source(s) of monetary or material support	Ministry of Science and Technology, China
Primary sponsor	The First Affiliated Hospital of Guangzhou Medical University
Secondary sponsor(s)	The First Affiliated Hospital of Guangzhou Medical University
Contact for public queries	Pixin Ran, MD, pxran@gzhmu.edu.cn
Contact for scientific queries	Yumin Zhou, MD, zhouyumin410@126.com
Public title	Long-term regular treatment of early COPD with Acetylcysteine effervescent tablets: a randomized, double-blind, placebo-controlled multicenter clinical study
Scientific title	Long-term regular treatment of early COPD with Acetylcysteine effervescent tablets: a randomized, double-blind, placebo-controlled multicenter clinical study
Countries of recruitment	China
Health condition(s) or problem(s) studied	Chronic obstructive pulmonary disease
Intervention(s)	Active comparator: *N*-acetylcysteine effervescent tablets (1200 mg per day)
	Placebo comparator: microcristallin cellulose (matching tablets containing no active ingredients)
Key inclusion and exclusion criteria	Aged 40–80 years old, male or female, community or outpatient
	Has respiratory symptoms (chronic cough, sputum, shortness of breath) and/or chronic obstructive pulmonary exposure risk factors (smoking, occupational exposure, indoor and outdoor air pollution, family history of chronic obstructive pulmonary disease, recurrent respiratory tract infection, low birth weight, and genetic factors, etc.)
	GOLD stages I–II, COPD: FEV_1_/FVC < 70% and FEV_1_ ≥ 50% predicted after 20 min with 400 μg of salbutamol inhalation
Study type	Interventional
	Allocation: randomized; intervention model: parallel assignment; masking: double blind
	Primary purpose: prevention
	Phase IV
Date of first enrolment	September 2017
Target sample size	1000
Recruitment status	Recruiting
Primary outcome(s)	The difference of FEV_1_ (including trough and peak) at 24 months from baseline and the number of acute exacerbations COPD within 24 months between two groups
Key secondary outcomes	Annual decline of FEV_1_, forced vital capacity (FVC) and FEV_1_/FVC (including trough and peak); duration, interval, and severity of COPD exacerbations

## Discussion

Early-stage COPD was defined as GOLD stage I–II COPD, according to the currently available clinical evidence [1]. Patients with early-stage COPD comprise 70.7% of all patients with the illness in China, and more than 60% of patients with early-stage COPD have no symptoms; therefore, most of these patients do not receive any treatment [3]. Our previous study (Tie-COPD) produced sufficient evidence that long-term using of a drug in patients with early-stage COPD has benefits on their lung function, COPD acute exacerbation rate, and life quality score [8].

Despite the relatively normal lung function value (FEV_1_/FVC, FEV_1_% of predict) and asymptomatic in patients with GOLD stage I–II COPD, oxidative/antioxidant imbalance and nonspecific airway inflammation, as well as relatively large amounts of airway secretions, are common in these patients [28, 29]. A large number of evidences have showed that the increase of oxidative stress plays an important role in the pathogenesis of COPD [30]. 4-Hydroxynonenal, thiobarbituric acid reactive substances/nitrotyrosine, and excessive oxidative markers can damage the airway epithelium, increase the apoptosis and autophagy, and finally lead to the destruction of airway wall elasticity and the formation of fibrosis, which is related to lung function decline and frequent exacerbation [31–33]. In these patients, the expression of reduced glutathione and endogenous antioxidants such as glutathione synthetase in the lung were significantly decreased [34]. Based on the above reasons, we believe that reducing oxidative stress and airway inflammation is a potential way in the prevention of severe COPD, especially in the early stage.

Oral NAC was a well-known, effective mucolytic agent that reduces sputum viscosity and elasticity with direct/indirect antioxidant and anti-inflammatory properties that may provide additional benefits in COPD, in whom the endogenous GSH pool is depleted [35]. NAC provides GSH precursors for active oxygen scavengers, inhibits redox-sensitive cell signal transduction and pro-inflammatory gene expression, and can effectively restore COPD cell oxidation/antioxidant balance and reduce the intensity of inflammatory response [14, 36, 37].

However, in previous studies, the clinical effects of regular NAC doses (400–800 mg/per day) in patients with chronic bronchitis or COPD have been inconsistent. Systematic reviews found that 800–1200 mg/day NAC could reduce COPD exacerbation rate versus placebo [38]. Conversely, in a 3-year randomized, double-blind, placebo-controlled study, researchers observed no significant benefit of 1000 mg/day NAC on the annual FEV_1_ decline rate and the number of acute exacerbations [39]. The inconsistent results may be due to the different doses of NAC used, and the difference in the population, and/or the use of inappropriate outcome parameters, such as the FEV_1_ decline rate, insensitive markers of small airway disease, and air retention [14, 38]. In the other two studies, despite a follow-up period of up to 3 years, the relatively small sample size (523 versus 238 patients) and the low dose of NAC treatment (600 mg/day versus 500 mg/day) did not achieve a significant result in reducing the exacerbation rate [39, 40]. Because a dose-effect association has been reported for NAC, we postulated that increasing the dose of NAC and extended follow-up time might improve outcomes.

In addition, previous studies also found that treatment with high-dose NAC (1200 mg per day) reduced rates of acute COPD exacerbations and hospital readmissions [17, 18]. Considering the large number of subgroup analyses used in these studies, we will also use subgroup analysis in our subsequent results if appropriate. Regarding the long-term treatment of early-stage COPD, the low price of NAC is also an important advantage, particularly in underdeveloped rural areas or urban low-income populations. Therefore, further longitudinal studies are required to confirm the clinical relevance and cost-effectiveness property of these discoveries.

We aimed to assess the beneficial effects of long-term treatment with high-dose NAC in patients with early-stage COPD regarding exacerbation rates and pulmonary function. The primary outcome measurement is FEV1 and the rate of acute COPD exacerbation. Discontinuations will increase with increasing study duration in long-term trials, and the rates are usually higher in the placebo group. The placebo group discontinuation rates in the ISOLDE, EUROSCOP, and UPLIFT studies ranged from 30 to 53%. In line with the UPLIFT protocol, we have estimated that the discontinuation rate is 35% in our study [41]. To ensure that it will be adequately powered to enable evaluation of the primary outcome, each group needs at least 500 random patients. Considering the need for acute exacerbations and lung function, the total required sample size of the study was 1000 patients. The majority of participants enrolled have no or extremely minor symptoms, and the mere presence of respiratory symptoms or gradually reduced lung function is an insufficient reason for patients to seek medical help [41]. In addition to the planned drug delivery and follow-up, we will provide health education, including counseling them to quit smoking, informing them of the benefits of early treatment, and providing other health advice. We will establish a good relationship with the research subjects and conduct regular telephone follow-up or Internet supervision to reduce the withdrawal rate.

In summary, because a dose-effect association has been demonstrated for NAC, this NAC-COPD trial will provide an opportunity to explore the effect of twice-daily NAC in patients with early-stage COPD disease. We postulated that an increased dose of NAC might lead to improved outcomes.

## Supplementary information

**Additional file 1.** List of research centers.
