# Supplementary material for: High-dose N-acetylcysteine for long-term, regular treatment of early-stage chronic obstructive pulmonary disease (GOLD I–II): study protocol for a multicenter, double-blinded, parallel-group, randomized controlled trial in China
Source: Trials. 2020 Sep 11;21:780. doi: 10.1186/s13063-020-04701-8 (PMC7488567; doi:10.1186/s13063-020-04701-8)
Supplement: Supplementary file 1 — Additional file 1. List of research centers. [file 13063_2020_4701_MOESM1_ESM.docx]

| **中文名字** | **name in English** |
| --- | --- |
| 广州医科大学附属第一医院 | The First Affiliate Hospital of Guangzhou Medical University |
| 复旦大学附属中山医院 | Zhongshan Hospital Affiliated to Fudan University |
| 浙江大学医学院附属邵逸夫医院 | Sir Run Run Shaw Hospital, Zhejiang University School of Medicine |
| 中国医科大学附属第一医院 | The First Affiliated Hospital of China Medical University |
| 温州医科大学附属第一医院 | The First Affiliate Hospital of Wenzhou Medical University |
| 复旦大学附属上海浦东医院 | Shanghai Pudong Hospital |
| 深圳市人民医院 | Shenzhen People's Hospital |
| 广西医科大学附属第一医院 | The First Affiliate Hospital of Guangxi Medical University |
| 北京大学第三医院 | Peking University Third Hospital |
| 暨南大学附属第一医院 | The First Affiliated Hospital of Jinan  University |
| 中南大学湘雅二医院 | The Second Xiangya Hospital of Central South University |
| 华中科技大学同济医学院附属同济医院 | Tongji Hospital, Tongji Medical College, Huazhong University of Science and Technology |
| 天津医科大学总医院 | Tianjin Medical University General Hospital |
| 浙江大学 | Zhejiang University |
| 上海交通大学医学院附属瑞金医院北院 | Ruijin Hospital North Campus, The Shanghai Jiao Tong University Medical School |
| 连平县人民医院 | Lianping People 's Hospital |
| 翁源县人民医院 | Wengyuan People 's Hospital |
| 湖南省第二人民医院 | The Second People's Hospital of Hunan Province |
| 湖南省人民医院 | Hunan provincial people's hospital |
| 惠州市第一人民医院 | Huizhou First Hospital |
| 广州市第一人民医院 | Guangzhou First People's Hospital |
| 广州医科大学附属第三医院 | The Third Affiliated Hospital of Guangzhou Medical University |
| 广州市荔湾中心医院 | Liwen Central Hospital of Guangzhou |
| 广东医科大学附属医院 | Affiliated Hospital of Guangdong Medical University |
